# Compartmentalized *Histoplasma capsulatum* Infection of the Central Nervous System

**DOI:** 10.1155/2015/581415

**Published:** 2015-06-14

**Authors:** Albert J. Eid, John D. Leever, Kathrin Husmann

**Affiliations:** ^1^Division of Infectious Diseases, University of Kansas Medical Center, 3901 Rainbow Boulevard, Kansas City, KS 66160, USA; ^2^Department of Radiology, University of Kansas Medical Center, 3901 Rainbow Boulevard, Kansas City, KS 66160, USA; ^3^Department of Neurology, University of Kansas Medical Center, 3901 Rainbow Boulevard, Kansas City, KS 66160, USA

## Abstract

*Background*. Histoplasmosis is a common fungal infection in the southeastern, mid-Atlantic, and central states; however, its presentation can be atypical. *Case Presentation*. We report a case of *Histoplasma capsulatum* infection presenting as slowly progressive weakness in the lower extremities, followed by the development of numbness below the midthoracic area, urinary incontinence, and slurred speech. Brain MRI showed leptomeningeal enhancement, predominantly linear, involving the basal cisterns, the brainstem, and spinal cord. Cerebrospinal fluid analysis showed lymphocytic pleocytosis. *Discussion*. CNS histoplasmosis is usually seen in patients with disseminated histoplasmosis. Isolated CNS histoplasmosis is rarely seen, especially in immunocompetent patients. *Conclusions*. Histoplasmosis should be considered in the differential diagnosis of patients experiencing slowly progressive neurological deficit.

## 1. Introduction

Histoplasmosis is a fungal infection caused by a dimorphic fungus called* Histoplasma capsulatum* throughout the world. In the United States, it is commonly seen in the southeastern, mid-Atlantic, and central states. Typically, patients become infected through inhalation of fungal spores causing an infection in the lungs. The infection might be asymptomatic but it could lead to flu-like syndrome, infection localized to the lungs, or disseminated infection. Herein, we report of case of* Histoplasma capsulatum* infection localized to the central nervous system (CNS).

## 2. Case Presentation

A 53-year-old woman with no significant past medical history presented with slowly progressive weakness in the lower extremities, new-onset numbness below the midthoracic area, urinary incontinence, and slurred speech. One year prior to presentation, she experienced left-sided transitory weakness involving the face and extremities. She was evaluated by multiple neurologists and her brain imaging studies were unrevealing. She later developed tremor in the upper extremities and she was suspected to have central tremor or Parkinson's disease. The patient never had fever, chills, or sweating; however, she had lost 30 pounds during the three-month period preceding her presentation.

The patient grew up on a dairy farm and played in silos. She is married; she lives and works in a farm in rural Kansas, where they grow corn and soy. She has no significant travel history. She denied having sick contacts or significant animal exposure. And she had no history of drug use, incarceration, or exposure to tuberculosis.

The patient first presented to an outside hospital. MRI of the brain and spine, as well as lumbar puncture (LP), was obtained. The MRI images showed leptomeningeal enhancement, predominantly linear, involving the basal cisterns, the brainstem, and spinal cord. Cerebrospinal fluid (CSF) analysis showed 4 rbc/mm^3^, 157 wbc/mm^3^ (55% lymphocytes, 35% neutrophils, and 10% monocytes), protein 362 mg/dL, and glucose 24 mg/dL. CSF Gram stain showed no organisms and AFB smear was negative. Bacterial culture was negative. CSF studies including cryptococcal antigen, human herpes simplex virus PCR, West Nile virus PCR, Lyme antibodies,* M. tuberculosis* (TB) PCR, and cytology were all negative. HIV screen, RPR, and QuantiFERON-TB Gold were negative as well. A CT of the chest, abdomen, and pelvis was unremarkable. The patient was treated with antibiotics and steroids and transferred to our hospital 8 days after her initial admission for further evaluation and treatment. At that time, her CSF fungal and mycobacterial cultures were pending.

**Figure 1:**
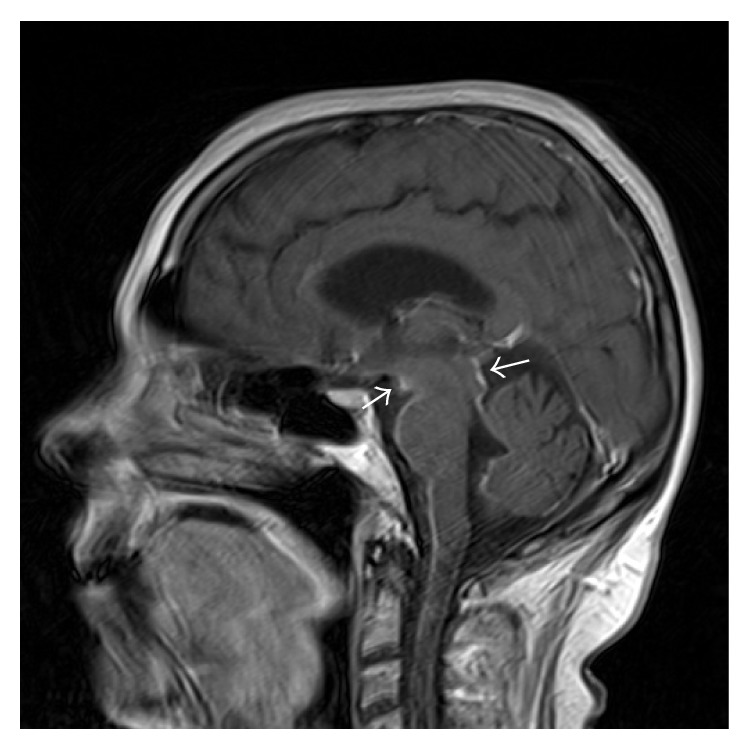
Sagittal T_1_ postcontrast image of the brain: linear leptomeningeal enhancement along the ventral brainstem, the tectum, and the upper cervical spinal cord.

**Figure 2:**
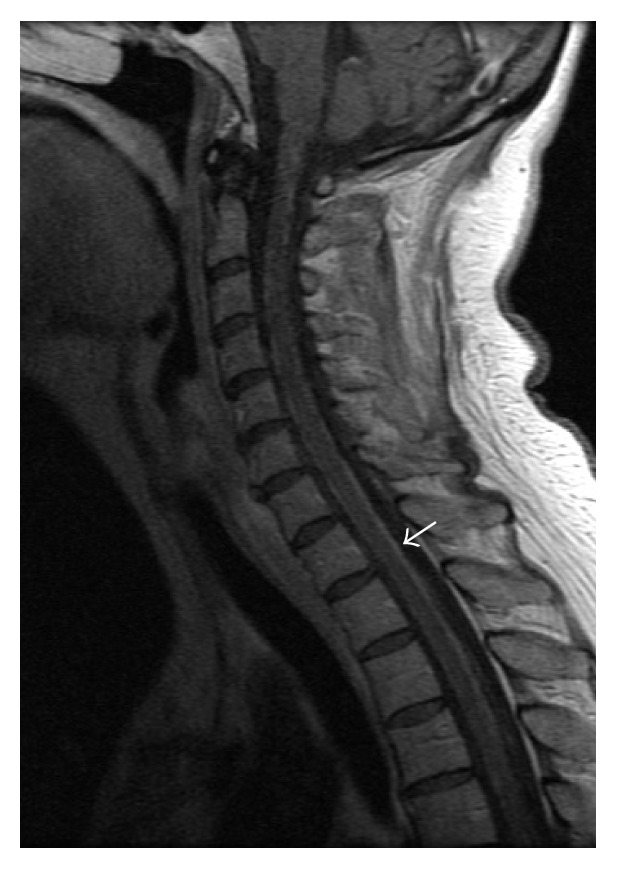
Sagittal T_1_ postcontrast image of the cervical spine: linear leptomeningeal enhancement along the cervical and visualized upper thoracic spinal cord.

**Figure 3:**
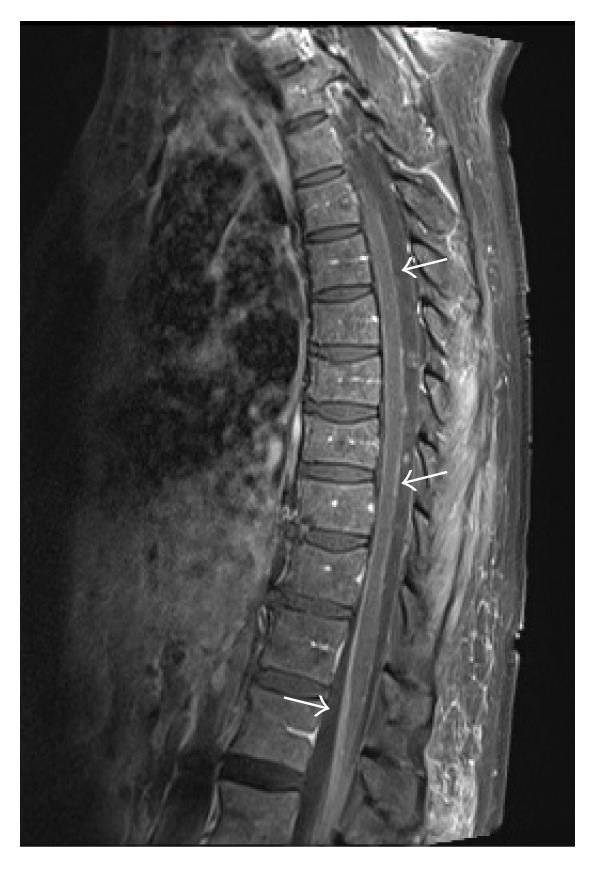
Sagittal T_1_ postcontrast fat-suppressed image of the thoracic spine: linear leptomeningeal enhancement of the thoracic spinal cord and cauda equina nerve roots.

After transfer to our facility, a second LP was performed, three days after the initial one. CSF analysis revealed 248 wbc/mm^3^ (77% lymphocytes, 6% neutrophils, and 17% monocytes), protein 340 mg/dL, and glucose 51 mg/dL. Routine bacterial culture was again negative. C-reactive protein and procalcitonin levels were normal. Repeat brain and spine MRI at our facility showed mild improvement of the meningeal enhancement (Figures 1, 2, and 3). Repeat CSF analysis two days later revealed 25 rbc/mm^3^, 150 wbc/mm^3^ (61% neutrophils, 23% lymphocytes, and 16% monocytes), glucose 14 mg/dL, and protein 587 mg/dL. The shift from lymphocytes to neutrophils was felt to be due to steroid therapy. The CD4 lymphocyte count was 372 (58.5%), despite treatment with steroids. The work-up at this point focused on ruling out a fungal infection since TB seemed unlikely.

Fungitell was negative. Serum* Histoplasma* antibody was positive by EIA; however, complement fixation and immunodiffusion assays were negative.* Histoplasma* urine antigen was 0.68 ng/mL.* Blastomyces* antibody was negative (immunodiffusion assay).* Blastomyces* urine antigen was 0.55 ng/dL.* Sporothrix* antibody was negative. CSF* Histoplasma* and* Blastomyces* antibodies were both negative. At this time, fluconazole, which was given initially for empiric antifungal therapy, was changed to liposomal amphotericin B. Repeat* Histoplasma* urine antigen was 0.71 ng/mL and* Blastomyces* urine antigen was 0.56 ng/dL. CSF* Histoplasma* and* Blastomyces* antigens were both positive, above the limits of quantification.

Last LP, two weeks later, showed further improvement of pleocytosis and protein level (60 wbc: 58% lymphocytes, 33% neutrophils, and 9% monocytes; glucose 54 mg/dL; protein 173 mg/dL). Repeat CSF* Histoplasma* and* Blastomyces* antibodies were again negative, while repeat CSF* Histoplasma* and* Blastomyces *antigens were both positive, above limits of quantification. Serum* Histoplasma* antigen was measured as well and came back positive, below limit of quantification (<0.4 ng/dL). Two CSF fungal and mycobacterial cultures done at the outside hospital remained negative. One of two CSF fungal cultures, obtained two days after admission to our facility, eventually grew* Histoplasma capsulatum* one month later. Four weeks after admission to our facility, the patient was discharged to a long-term acute care hospital to continue treatment. She received 6 weeks of treatment with liposomal amphotericin B followed by oral voriconazole* with minimal improvement of her weakness during the first two months of treatment*. Patient was lost to follow-up afterwards.

## 3. Discussion

CNS histoplasmosis is usually seen as a manifestation of disseminated histoplasmosis, at the time of presentation or during disease relapse after discontinuation of antifungal therapy [1]. Isolated CNS histoplasmosis, however, is rarely seen, especially in immunocompetent patients [2]. Subacute or chronic meningitis is the most common manifestation of CNS histoplasmosis, occurring in 10–30% of patients with disseminated histoplasmosis [3]. Other manifestations include granulomatous lesions (histoplasmoma), embolism from* Histoplasma* endocarditis, and cerebral vasculitis with focal ischemia [4]. Even though CNS histoplasmosis is more prevalent in immunocompromised patients, around 20–30% of patients with this disease have intact immune system [2].

The diagnosis of CNS histoplasmosis can be challenging. None of the diagnostic tests has high sensitivity, which mandates the concomitant use of multiple tests in order to establish the diagnosis. Even though CSF fungal culture is the most specific test, its sensitivity can be as low as 25%. Large volume of CSF is required to increase the yield. Therefore, at least 10 mL of CSF should be cultured, and cultures should be repeated when additional CSF samples are obtained.* Histoplasma capsulatum* can take up to 6 weeks to grow in culture, which significantly delays the diagnosis and possibly the treatment. In early reports, the sensitivity of* Histoplasma* antigen detection in the CSF was 38% in all patients and 67% in patients with AIDS. However, the current antigen assay is likely more sensitive than the older one [5]. Cross-reaction is seen with blastomycosis, paracoccidioidomycosis, and* Penicillium marneffei.* False-positive results can be caused by interfering substances such as human anti-rabbit antibodies, rheumatoid factor, or heterophil antibodies. However, the specificity of the CSF* Histoplasma* antigen remains high (96%). Anti-*Histoplasma* antibodies in the CSF have good sensitivity (80–89%) and specificity (83%) and can be a helpful adjunct to establish the diagnosis. Serum anti-*Histoplasma* antibodies are positive in 86% of immunocompetent patients, 80% of non-AIDS immunosuppressed patients, and 67% of patients with AIDS. Interestingly enough, in our immunocompetent patient, both CSF and serum anti-*Histoplasma* antibodies were repeatedly negative. Urine* Histoplasma* antigen has 71% sensitivity and 99% specificity in patients with* Histoplasma* meningitis, while serum antigen has only 38% sensitivity but ~98% specificity [5]. Higher antigen levels in the CSF, as seen in our patient, are suggestive of antigen production in the CNS, rather than passive diffusion from the serum due to disruption of the blood-brain barrier or contamination of the CSF with blood due to traumatic LP [2].

The optimal treatment for CNS histoplasmosis is not known. However, the recommended treatment is liposomal amphotericin B (5 mg/kg daily for a total of 175 mg/kg over 4–6 weeks) followed by itraconazole (200 mg 2 or 3 times daily) for at least 1 year and until resolution of CSF abnormalities, including negative* Histoplasma* antigen [6]. Voriconazole has been used in clinical practice, however, due to better CNS penetration.

## References

[1] Threlkeld Z. D., Broughton R., Khan G. Q., Berger J. R. (2012). Isolated Histoplasma capsulatum meningoencephalitis in an immunocompetent child. *Journal of Child Neurology*.

[2] Ramireddy S., Wanger A., Ostrosky L. (2012). An instructive case of CNS histoplasmosis in an immunocompetent host. *Medical Mycology Case Reports*.

[3] Wheat L. J., Kohler R. B., Tewari R. P., Garten M., French M. L. V. (1989). Significance of Histoplasma antigen in the cerebrospinal fluid of patients with meningitis. *Archives of Internal Medicine*.

[4] Wheat L. J., Batteiger B. E., Sathapatayavongs B. (1990). Histoplasma capsulatum infections of the central nervous system: a clinical review. *Medicine*.

[5] Wheat L. J., Musial C. E., Jenny-Avital E. (2005). Diagnosis and management of central nervous system histoplasmosis. *Clinical Infectious Diseases*.

[6] Wheat L. J., Freifeld A. G., Kleiman M. B. (2007). Clinical practice guidelines for the management of patients with histoplasmosis: 2007 update by the Infectious Diseases Society of America. *Clinical Infectious Diseases*.

